# International Consensus (ICON): allergic reactions to vaccines

**DOI:** 10.1186/s40413-016-0120-5

**Published:** 2016-09-16

**Authors:** Stephen C. Dreskin, Neal A. Halsey, John M. Kelso, Robert A. Wood, Donna S. Hummell, Kathryn M. Edwards, Jean-Christoph Caubet, Renata J. M. Engler, Michael S. Gold, Claude Ponvert, Pascal Demoly, Mario Sanchez-Borges, Antonella Muraro, James T. Li, Menachem Rottem, Lanny J. Rosenwasser

**Affiliations:** 1 Division of Allergy and Clinical Immunology, Department of Medicine, University of Colorado Denver School of Medicine, Aurora, CO USA; 2 Institute for Vaccine Safety, Department of International Health, Johns Hopkins Bloomberg School of Public Health, Baltimore, MD USA; 3 Division of Allergy, Asthma, and Immunology, Scripps Clinic, San Diego, CA USA; 4 The Division of Pediatric Allergy and Immunology, Department of Pediatrics, Johns Hopkins University School of Medicine, Baltimore, MD USA; 5 Division of Pediatric Allergy, Immunology, and Pulmonary Medicine, Department of Pediatrics, Vanderbilt University School of Medicine, Nashville, TN USA; 6 Division of Pediatric Infectious Diseases, Department of Pediatrics, Vanderbilt University School of Medicine, Nashville, TN USA; 7 Department of Pediatrics, University Hospitals of Geneva and Medical School, University of Geneva, Geneva, Switzerland; 8 Department of Medicine and Pediatrics, Uniformed Services University of the Health Sciences, Allergy-Immunology-Immunization, Walter Reed National Military Medical Center, Bethesda, MD USA; 9 Disipline of Paediatrics, School of Medicine, University of Adelaide, Adelaide, South Australia Australia; 10 Department Paediatrics, Pulmonology and Allergy service, Necker-Enfants Malades Hospital, 149 rue de Sèvres, 75015 Paris, France; 11 Département de Pneumologie et Addictologie, Hôpital Arnaud de Villeneuve - University Hospital of Montpellier, 34295 Montpellier cedex 05 – FRANCE and Sorbonne Universités, UPMC Paris 06, UMR-S 1136 INSERM, IPLESP, Equipe EPAR, 75013 Paris, France; 12 Allergy and Clinical Immunology Department, Centro Médico Docente La Trinidad, Caracas, Venezuela; 13 Food Allergy Referral Centre Department of Women and Child health, University of Padua, Padua, Italy; 14 Division of Allergic Diseases, Mayo Clinic, Rochester, MN USA; 15 Allergy Asthma and Immunology, Emek Medical Center, Afula, and the Rappaport Faculty of Medicine, Technion- Israel Institute of Technology, Haifa, Israel; 16 Allergy-Immunology Division, Children’s Mercy Hospital and the University of Missouri-Kansas City School of Medicine, Kansas City, MO USA

**Keywords:** Allergy, Allergic reactions, Anaphylaxis, Causality, Components, International, Consensus, Vaccine

## Abstract

**Background:**

Routine immunization, one of the most effective public health interventions, has effectively reduced death and morbidity due to a variety of infectious diseases. However, allergic reactions to vaccines occur very rarely and can be life threatening. Given the large numbers of vaccines administered worldwide, there is a need for an international consensus regarding the evaluation and management of allergic reactions to vaccines.

**Methods:**

Following a review of the literature, and with the active participation of representatives from the World Allergy Organization (WAO), the European Academy of Allergy and Clinical Immunology (EAACI), the American Academy of Allergy, Asthma, and Immunology (AAAAI), and the American College of Allergy, Asthma, and Immunology (ACAAI), the final committee was formed with the purpose of having members who represented a wide-range of countries, had previously worked on vaccine safety, and included both allergist/immunologists as well as vaccinologists.

**Results:**

Consensus was reached on a variety of topics, including: definition of immediate allergic reactions, including anaphylaxis, approaches to distinguish association from causality, approaches to patients with a history of an allergic reaction to a previous vaccine, and approaches to patients with a history of an allergic reaction to components of vaccines.

**Conclusions:**

This document provides comprehensive and internationally accepted guidelines and access to on-line documents to help practitioners around the world identify allergic reactions following immunization. It also provides a framework for the evaluation and further management of patients who present either following an allergic reaction to a vaccine or with a history of allergy to a component of vaccines.

## Introduction

Routine immunization, one of the most effective public health interventions, has effectively reduced death and morbidity due to a variety of infectious diseases [1, 2]. Very rarely, allergic reactions to vaccines occur, and can be life threatening [3–6]. Estimates of allergic reactions to vaccines including immediate hypersensitivity reactions, range from 1 in 50,000 to 1 in 1,000,000 doses [7–9]. The most concerning of these, anaphylaxis, has been estimated to occur at a rate of approximately one per 100,000 to one per 1,000,000 doses for most commonly administered vaccines [8, 10, 11] (B)^1^. The true rate of allergic reactions is unknown because most reactions are not reported.

Allergic reactions need to be distinguished from clinical manifestations that occur coincidental to vaccination (e.g. becoming anxious), vasovagal responses, local injection-site reactions (either immediate or delayed), and the oculorespiratory syndrome (ORS). Allergic reactions are generally immediate and IgE-mediated. Symptoms vary from relatively minor cutaneous signs and symptoms (erythema and itching) to multisystem effects (anaphylaxis) that can include the cutaneous, respiratory, gastrointestinal, and/or cardiovascular systems. Allergic reactions can be due to allergy to vaccine antigens (portions of organisms or toxoids), residual media used to grow organisms, stabilizers, preservatives, or other excipients [6] (B). Given the increasing prevalence of allergic disease throughout the world [12–15], it is not surprising that there are increasing concerns about possible allergic reactions following vaccines and concerns about vaccine components.

Patients may have clinical complaints that occur immediately subsequent to administration of a vaccine that may or may not be compatible with an allergic reaction, but nonetheless have significant impact on the patient’s perception of vaccines and their willingness to undergo further vaccination. In addition, patients may have complaints that have a delayed onset relative to having received a vaccine that raise concerns about delayed allergic or other immunologic reactions to vaccine components.

A variety of very useful documents in the literature have addressed many of these concerns [3–6], but none have addressed all of these issues or have presented an international consensus. For this reason, the World Allergy Organization (WAO) initiated an effort to publish this International CONsensus (ICON) on allergic reactions to vaccines. The intent of this document is to identify themes that commonly occur in a large variety of settings and to provide a comprehensive reference for a systematic approach to the problems related to allergic reactions to vaccines.

Following the above introduction (Part I), this document is organized to first describe our methodology, process, and to provide definitions (Part II). In subsequent sections, we review allergic reactions to specific vaccines (Part III) and then allergic reactions to components of vaccines (Part IV). Finally, we address the recommended approach to the patient with a history of an allergic reaction to vaccines (Part V) and to the patient with a history of an allergic reaction to an exogenous substance (e.g. food, drug, or latex) that may be found in a vaccine or its packaging (Part VI). In closing, we address unmet needs and offer suggestions for future research (Part VII). Since some specific vaccines are discussed from several points of view, some redundancy is unavoidable.

## Methodology

### Participants

Under the auspices of WAO, a working committee was formed, consisting of Drs. Rosenwasser, Dreskin, and Halsey. Following a review of the literature, and with the active participation of representatives from the European Academy of Allergy and Clinical Immunology (EAACI), the American Academy of Allergy, Asthma, and Immunology (AAAAI), the American College of Allergy, Asthma, and Immunology (ACAAI), the final committee was formed with the purpose of having members who represented a wide-range of countries, had previously worked on vaccine safety, and included both allergist/immunologists as well as vaccinologists.

### Process

Following email contact, a conference call was convened during which participants agreed to write or to help write specific parts of this ICON, relying heavily on previously published ICONs as well as a practice parameter on adverse reactions to vaccines and other reviews of allergic reactions to vaccines [3–6]. The first draft of a complete document was then compiled by Drs. Dreskin and Halsey and subsequently sent to all participants for final editing. A second conference call was then held to discuss differences in opinion. Then a final draft was sent to participants for their review. This draft was then sent to an independent committee (chosen on the basis of participating in previous ICONs) and their comments circulated back to the committee for decision regarding further alteration. A final document was then approved by the Board of Directors of the sponsoring organizations.

## Definitions

### Immediate reactions that are not allergic (Immediate non-allergic reactions)

Local, injection site reactions (swelling, redness, and/or soreness) and constitutional symptoms, especially fever, are common after the administration of many vaccines and are not contraindications to subsequent vaccination [16] (D).

### Immediate allergic reactions

Immediate hypersensitivity or allergic reactions to vaccines are rare but potentially serious adverse events that require investigation and understanding of the associated risks in order to properly counsel patients regarding the risk versus benefit ratio for the administration of future vaccines. In this document, “allergy” will be used interchangeably with “immediate hypersensitivity” and “IgE-mediated reaction” as descriptors to denote a presumed underlying IgE-mediated immune mechanism for an adverse event. We use the term “immediate” to distinguish these allergic reactions from those that may be mediated by antibodies other than IgE or by T cells (commonly seen in immunologic reactions to drugs).

### Limited immediate allergic reactions

Allergic reactions to vaccines may be mild and limited in the scope of symptoms and involvement of organ systems, or even localized to the site of vaccine administration. Thus, typical signs of an allergic reaction may include bronchoconstriction, rhinoconjunctivitis, gastrointestinal symptoms, and/or characteristic skin lesions such as generalized urticaria and/or angioedema [17], occurring as a sole sign with an onset within minutes and less than 4 h post-vaccination [4] (D).

### Anaphylaxis

#### Definition of Anaphylaxis

Anaphylaxis is the most severe form of an IgE-mediated reaction, encompassing a spectrum of symptoms and involvement of several organ systems. For the majority of instances, anaphylaxis occurs within minutes following an exposure to an allergen. The International Consensus on (ICON) Anaphylaxis published in 2014 reviewed definitions proposed by WAO; the Joint Task Force on Practice Parameters, representing the AAAAI, the ACAAI, and the Joint Council of Allergy, Asthma, and Immunology (JCAAI); and the EAACI. In this consensus document, all organizations have agreed upon the concept that anaphylaxis is a “serious, generalized or systemic, allergic or hypersensitivity reaction that can be life-threatening or fatal” [18] (D).

The National Institutes of Allergy and Infectious Diseases (NIAID) / Food Allergy and Anaphylaxis Network (FAAN) criteria developed in 2006 by an NIH meeting of experts in the fields of allergy and immunology defined anaphylaxis as one of three scenarios: 1) The acute onset of an illness within minutes or hours with involvement of: skin and/or mucosa (pruritus, flushing, hives, angioedema), and either respiratory compromise (dyspnea, wheeze/bronchospasm, decreased peak expiratory flow, stridor, hypoxemia) OR decreased blood pressure/end organ dysfunction (collapse, syncope, incontinence) 2) Two or more of the following that occur rapidly after exposure to a *likely* allergen for that patient: skin and/or mucosa; respiratory compromise; decreased blood pressure/end organ dysfunction; persistent GI symptoms (vomiting, crampy abdominal pain, diarrhea) 3) The following within minutes or hours after exposure of a *known* allergen for that patient: decreased blood pressure [19] (D).

Alternative criteria include those developed by the Brighton Collaboration Working Group for case definitions [20] (D). These criteria are not intended to distinguish differing levels of severity of anaphylaxis, but instead denote different levels of diagnostic certainty, as the definition is used primarily for epidemiologic studies. A Level 1 case definition has the highest level of diagnostic certainty, with progressively lower certainty for levels 2 and 3, respectively. Because these levels do not directly define severity, it is possible for a very severe clinical event to be classified as a level 2 or 3, based on the available information. Furthermore, appropriate rapid treatment of an incipient immediate hypersensitivity reaction with intramuscular epinephrine may modulate the severity of the reaction [18] (D).

Although most episodes of anaphylaxis involve cutaneous symptoms of urticaria and/or angioedema, this is not universally the case. Skin and/or mucosal signs may be absent in 10–20 % of all episodes, and hypotension in infants often remains unrecognized. Unique aspects of anaphylaxis in infants, including behavioral changes and challenges regarding recognition of cardiovascular signs has recently been reviewed [21]. In general, underreporting of anaphylaxis is likely common [22] (D).

Most episodes of anaphylaxis occur with a sudden onset and rapid progression [23] (D). Biphasic reactions are also described, in which an initial clinical presentation resolves with or without treatment, to be followed later (up to 72 h) by a recurrence [24, 25] (D). Protracted anaphylaxis (lasting up to several days without resolution) has also been described, but is uncommon and the literature consists only of case reports or small series [26] (D). Protracted anaphylaxis has been reported following administration of vaccines [11] (D).

It is therefore not possible to assign a strict time frame (time from exposure to onset of symptoms) upon the definition of anaphylaxis in relation to a potential triggering event, such as an immunization. The AAAAI and ACAAI Joint Task Force on Practice Parameters advised considering events with onset within 4 h of vaccine administration as possibly consistent with anaphylaxis [4] (D). Guidelines from the EAACI note that symptoms and signs of anaphylaxis usually occur within 2 h of exposure to the allergen and this is even faster following exposure to parenteral medications or insect stings (venom) [27] (D). A review of a registry of anaphylactic reactions in the UK found that the median time to respiratory or cardiac arrest for reactions to venom (a parenteral exposure) was 15 min, with the longest interval being 120 min [28] (D).

The differential diagnosis of, and the potential triggers for, anaphylaxis must be considered whenever an episode appears to coincide with vaccine administration, since assessing the likelihood of causality (i.e. the vaccine causing anaphylaxis) is heavily dependent upon there being no alternative cause that can be implicated (Table 1) [29] (D).

**Table 1:** Differential diagnosis of anaphylaxis

Anaphylaxis due to other allergenic or external exposures:	
Food (including scombroidosis), medication, insect venom, exercise, heat, cold, idiopathic.	
Anaphylaxis due to excess histamine production:	
Systemic mastocytosis, mast cell activation syndromes.	
Flushing syndromes	
Red man syndrome (vancomycin or other medication), carcinoid, postmenopausal, alcohol-related, vasoactive-peptide tumors (e.g. pancreatic VIPoma, medullary thyroid carcinoma).	
Miscellaneous	
Vasovagal episodes, panic attacks, vocal cord dysfunction, C1 inhibitor deficiency syndromes (hereditary and acquired), pheochromocytoma, neurologic process (seizure/stroke), cardiovascular process (myocardial infarction, embolism), capillary leak syndrome, dehydration, hypoglycemia.	

The WAO has suggested removing the term “anaphylactoid” from use, and this is supported by the most recent update of anaphylaxis published by the Joint Task Force on Practice Parameters, representing the AAAAI, the ACAAI, and the JCAAI [29] (D). Historically, this term referred to the same syndrome as anaphylaxis that was caused by immune mechanisms, but not involving serum IgE specific for an allergen. Other non-IgE-mediated immunologic mechanisms may cause anaphylaxis. For example, IgG-mediated and immune complex-mediated anaphylaxis has been reported for certain medications and biologic agents [30] (D), and non-immune activation of mast cells and basophils may occur [31]. However, it is now recognized that because anaphylaxis is a syndrome, with specific clinical features, and because the underlying immune mechanisms cannot easily be ascertained at the time of the event, it is essential to treat all episodes that fall into this category the same. Non-IgE-mediated events will not be discussed in this document except as they may be considered in the differential diagnosis for an adverse event (Table 1).

The CDC and FDA supported passive surveillance system, Vaccine Adverse Event Reporting System (VAERS), uses the term “serious” to include death, hospitalization or prolongation of hospitalization, persistent or significant disability/incapacity, or is life threatening. In this document, we use “serious” throughout the document in the same manner as clinicians use the term and not precisely as defined by VAERS.

#### Differential diagnosis of anaphylaxis

There are a number of immediate adverse events following immunization that could be misdiagnosed as anaphylaxis. For example, sudden events such as syncope following immunization may be confused with anaphylaxis. Many of these adverse events occur more commonly than vaccine related anaphylaxis and alternative diagnoses should be considered when a case definition for anaphylaxis is not met.

Anaphylaxis (all causes) usually presents with characteristic and predictable multi-system findings; less than 10 % of episodes present with sudden onset of hypotension (manifest as collapse/unresponsiveness) without concomitant respiratory manifestations and/or cutaneous signs (erythema, urticaria or angioedema). When sudden collapse or acute respiratory symptoms occur without skin changes following immunization, anaphylaxis should be considered.

Adverse events, other than anaphylaxis, that commonly result in sudden collapse and unresponsiveness following immunization include, in an infant, a Hypotonic Hyporesponsive Episode (HHE). HHE is characterized by the sudden onset of unresponsiveness, hypotonia and pallor, and usually presents 1-to-6 h after immunization [32]. Cardiovascular compromise and specifically hypotension does not occur in HHE. Vasovagal syncope can occur at all ages and is now a frequently reported adverse event since adolescents are at increased risk and adolescent vaccination is widely promoted in some countries [33]. In vasovagal syncope, hypotension is transient and associated with bradycardia rather than tachycardia as would occur typically in anaphylaxis. Sudden unresponsiveness due to a febrile seizure following immunization is frequently associated with tonic-clonic motor movements and no cardiovascular compromise.

Acute respiratory distress with cough and stridor may occur following minor unintentional aspiration of an oral vaccine (oral polio or rotavirus vaccine) and may be mistaken for anaphylaxis. In very rare instances, an error in vaccine administration may result in acute collapse and unresponsiveness that is neither HHE or vasovagal syncope. For example, inadvertent injection of a medication (for example a muscle relaxant) rather than the vaccine or following injection of staphylococcal toxin from a contaminated vial leading to Toxic Shock Syndrome [34, 35].

The oculo-respiratory syndrome (ORS) is defined by the onset within 24 h of immunization of at least one of the following symptoms: bilateral red eyes or respiratory symptoms (cough, sore throat, difficulty swallowing, wheeze, difficulty breathing, chest tightness) or facial edema [36]. The condition was primarily associated with two Influenza vaccines which contained high amounts of aggregated viron particles that triggered the signs and symptoms that were not a Type I hypersensitivity reaction [37, 38]. Refinements in manufacturing resulted in marked reductions in the incidence of this problem. Although ORS symptoms usually begin several hours after vaccination [37], making the symptoms less likely to be due to immediate hypersensitivity, a detailed assessment, including skin testing, may be required to differentiate ORS from anaphylaxis.

#### Epidemiology of anaphylaxis

Anaphylaxis following vaccine administration is a rare event, estimated to occur at a rate of approximately 1 per million vaccine doses (B) [8]. Fatalities are exceedingly rare [39] (D). More frequent acute events that occur following administration of vaccines may be confused with anaphylaxis, including vasovagal reactions, panic (anxiety) attacks, and vocal cord dysfunction (Table 1). The correct diagnosis is critically dependent upon obtaining essential details in the history surrounding the event [40] (D). This may provide details of exposure to allergens other than vaccines, or may discern other possible alternative diagnoses (Table 1). An accurate history is also essential to confirm that the timing of the event (onset in minutes to 4 h, see above) is compatible with the biologic plausibility of anaphylaxis to a vaccine.

### Delayed reactions

Rarely, delayed-type hypersensitivity to a vaccine constituent (e.g. aluminum) may cause an injection site nodule, but this is not usually a contraindication to subsequent vaccination. Delayed anaphylaxis (onset 3 to 6 h after exposure) is a concept that has recently been well described but in the context of individuals that have been bitten by the lone star tick and then develop IgE to a component of red meat, galactose-alpha-1, 3-galactose (alpha-gal) [41]. One patient with alpha gal allergy has safely received a gelatin containing vaccine and the authors found no documented published reports of alpha gal allergy resulting in anaphylaxis following vaccines in other patients with alpha gal allergy [42]. Of note, the route of exposure with red meat (ingestion) is different from the route of administration of vaccines (parenteral) and a delayed response due possibly to metabolic processes is more likely. Thus, vaccine-related allergic reactions including anaphylaxis should occur more quickly than seen in patients with allergy to red meat. Any vaccine-related reactions occurring more than 4 h after administration of a vaccine are unlikely to be immediate hypersensitivity reactions [43].

### Other immunologic reactions

Possible non-IgE-mediated reactions to vaccines include a broad range of adverse events following immunization (AEFI) and are commonly listed on the package inserts. These include mild fever and local reactions to life threatening infections following live vaccines inappropriately given to patients with immune deficiencies. Known side-effects from vaccines are detailed on the relevant Centers for Disease Control (CDC) website [44]. The Global Vaccine Safety Initiative addressing comprehensive AEFI considerations is reviewed on the WHO website [45].

### Association versus causality

Adverse events that temporally follow immunization are often attributed to the vaccine, suggesting a causal link to a component of the vaccine or to the immunologic response to the vaccine. Many AEFI are coincidental events that are falsely attributed to vaccines because of the temporal association. Causality, particularly with rare events and/or complex multifactorial disorders with documented delays in diagnosis (e.g. narcolepsy**)**, can be difficult to prove or disprove. For these reasons, careful analyses of many AEFIs have failed to substantiate or rule out a causal association.

Reports of temporal associations do not provide support for causality, but may indicate a need for future careful study to collect supportive data for a causal hypothesis [46]. Controlled trials are useful for identifying an association between administration of a vaccine and common events that may occur within a relatively short time period following an immunization, but are not as helpful for events that occur rarely or are significantly delayed in onset. In the case of hypersensitivity reactions, especially anaphylaxis, which has an abrupt and sudden onset usually within minutes following the allergenic exposure, a causal relationship is assumed when there are no other exposures such as food that could have caused the adverse event. Even when such a temporal association is made, other evidence should be sought when possible to identify the allergen responsible and to confirm the absence of evidence that points to an alternate cause.

The Causality Working Group of the Clinical Immunization Safety Assessment network have recently published an algorithm to help guide the systematic evaluation of an AEFI to help determine further steps to care for specific patients [47] (D) and to provide an assessment tool to help evaluate causality [48] (D).

In addition, the Institute of Medicine (IOM) engaged committees of experts to review the epidemiologic, clinical and biological evidence regarding causal associations with adverse health effects and specific vaccines covered by the U.S. Vaccine Injury Compensation Program (VICP). The latest review, titled “Adverse Effects of Vaccines: Evidence and Causality”, is available online [49]. The report classifies the evidence regarding many potential associations between specific vaccines and specific adverse events as a) convincingly supporting, b) favoring a causal relationship or c) rejecting a causal relationship. For a large number of other potential associations, it was determined “Evidence is inadequate to accept or reject a causal relationship”.

International efforts to support global standardization of case definitions for further research on adverse events are summarized by the Brighton Collaboration and provide an evolving profile of the questions raised about adverse events possibly linked to vaccines [50]. Further discussion of the spectrum of AEFI-vaccine questions is beyond the scope of this review.

## Allergic reactions to specific vaccines

In the sections that follow the allergic reactions to several of the commonly administered vaccines will be reviewed.

### Diphtheria, Tetanus, acellular Pertussis (DTaP) vaccine

Hypersensitivity reactions to diphtheria, tetanus and pertussis toxoid containing vaccines are very rare. Most reports concern injection site reactions, and among these are delayed hypersensitivity to aluminum included in the vaccine as an adjuvant [51–53] (C for Jackson, D for Beveridge and for Bergfors). Jackson et al. reported post-vaccination rates of fever, seizures, medically-attended injection site reactions, and urticaria responses within 7 days of immunization with DTaP between 1997 and 2000 in a retrospective population of patients from the Group Health Cooperative, an health-maintenance organization based in Seattle, WA with an enrollment of >360,000 persons, including approximately 27,000 children under age 7 years [54] (C). They found an overall rate of 3.9 episodes of urticaria reported per 10,000 doses of vaccine distributed. There was a trend toward increased rate of urticarial reactions with successive administration of the first four doses, with the highest rate of 8.9 cases per 10,000 for dose number 4 administered at age 15 months. The rate then fell to 2.5 for dose number 5, administered at age 5 years. Of the total of 30 visits for rashes diagnosed as consistent with urticaria, four presented on the day of vaccination, 11 had onset from days 1 through 3 post-vaccination, and 15 had onset from days 5 through 7 post-vaccination. No episodes of anaphylaxis were reported [54]. Cheng et al. evaluated events suspected or reported to be anaphylaxis in Australian children (<18 yo) from 2007 to 2013 and estimated a rate of 0.36 cases per 100,000 doses for DTaP [11].

DTP vaccines prior to 1997, but not since, contained traces of gelatin, either poorly hydrolyzed bovine gelatin as reported in Japan, or hydrolyzed porcine gelatin. Some have speculated that this may have resulted in gelatin sensitization in select populations [55, 56] (D), but this has not been established as a cause for allergic reactions to DTaP, and others refute this connection [57] (D)**.**

### Influenza vaccine

Influenza vaccines are unique in that the vaccine formulation changes often, based upon the strains of influenza projected to circulate in the upcoming season. In 2009, in response to a global influenza pandemic, a monovalent vaccine for pandemic influenza (H1N1) was introduced separately from the recommended seasonal influenza vaccine. Subsequently the H1N1 pandemic vaccine component has been included as the H1N1 component of the seasonal vaccine. Most influenza vaccines marketed currently are produced in embryonated chicken eggs, and therefore contain small amounts of egg proteins, most notably ovalbumin, the amounts of which may vary by vaccine manufacturer and vaccine lot. A new recombinant influenza vaccine produced in a baculovirus-insect cell system (Flublok®) is currently licensed in the United States only for recipients aged 18–49 years. This vaccine has reduced immunogenicity in children when compared with standard egg-grown vaccines [58] (B). Another recently licensed influenza vaccine is produced in cell culture (Flucelvax®) [59].

A previous severe allergic reaction to influenza vaccine, regardless of the component suspected of being responsible for the reaction, requires evaluation before future receipt of the vaccine in question or an alternative vaccine.

A 2014 publication reviewed the 2011 report of the Institute of Medicine concerning the adverse effects of childhood vaccines and also updated the findings by searching the following databases: DARE (Database of Abstracts of Reviews of Effects), the Cochrane Database of Systematic Reviews (Cochrane Reviews), Cochrane Central Register of Controlled Trials (CENTRAL), PubMed, Excerpta Medica dataBASE (EMBASE), Cumulative Index to Nursing and Allied Health (CINAHL), Toxicology Literature Online (TOXLINE), Advisory Committee on Immunization Practices (ACIP) statements, and vaccine package inserts. In this extensive review of adverse events reported following influenza immunization, anaphylaxis was not commented upon, due to its infrequent occurrence [60] (D).

An analysis of reports to VAERS of reactions following the 2009 administration of the H1N1 monovalent influenza vaccine revealed an overall rate of 10.7 immediate hypersensitivity reactions per million vaccine doses distributed, with a 2-fold higher rate for live attenuated vaccine as compared to inactivated vaccine [61]. The rate of anaphylaxis was 0.8 per million doses, with no significant differences by type of vaccine or manufacturer. A Vaccine Safety Datalink study (VSD) covering influenza vaccine immunizations in the 2012–2013 influenza season failed to find an increase in risk of anaphylaxis related to influenza vaccine administration compared with historical controls, adjusting for age and site. Among over 3.3 million first doses of Inactivated Influenza Vaccine (IIV) administered to individuals age 6 months and older, there were seven cases of reported anaphylaxis and no cases of anaphylaxis reported among 232,406 first doses of Live Attenuated Monovalent Influenza Vaccine (LAMV) administered. This compared with 18 cases of anaphylaxis per 11.2 million doses IIV and two cases per 338,000 doses of LAMV in the historical seasons of 2005–2006 through 2009–2010 [62](C). Similarly, a review of reported adverse events following immunization for pandemic monovalent H1N1 vaccine in Latin American and the Caribbean in the 2009–2010 season reported anaphylaxis as 7.6 % of the 1000 events supposedly attributable to vaccines and immunizations, representing a rate of 0.53 cases per million doses (0.41–0.64, 95 % CI). Of these, 45/76 cases occurred in age group 18–59 years, and 14 occurred in those under age 2 years [63] (D).

Egg allergy does not appear to impart an increased risk of an anaphylactic reaction to immunization with either inactivated or live attenuated influenza vaccines currently available in the United States and Europe (discussed in detail below under the heading "Approach to the patient with possible allergies to foods or other materials that may also be components of vaccines or vaccine packaging"). Although cases of immediate hypersensitivity reaction such as urticaria may occur, they appear to be no more common in egg-allergic than non-egg-allergic vaccine recipients [64, 65] (D). A review of articles in 2008 relating to allergic reactions, asthma, or food allergy yielded a number of cases of anaphylaxis following LAIV, although no evidence was found of a direct causal relationship to egg allergy [66] (D). Egg proteins are not the only component of influenza vaccines that may be responsible for an immediate allergic reaction.

The preservative, thimerosal, has been rarely implicated as causing allergic reactions to influenza vaccines but has not clearly demonstrated to be responsible [67] (D). Latex may be present in the rubber stopper of some vaccine vials and plungers in some prefilled syringes, but this appears to be a very rare issue for latex-sensitive individuals [68] (C). IgE directed toward the influenza component itself is rarely implicated in hypersensitivity reactions [4, 43] (D). Other allergic or hypersensitivity reactions described following immunization with influenza vaccine may not be IgE-mediated [43].

The United States joint task force on Practice Parameters of the AAAAI and ACAAI states that “special precautions regarding medical setting and waiting periods after administration of IIV to egg-allergic recipients beyond those recommended for any vaccine are not warranted." [139] (D). The Canadian National Advisory Committee on Immunization (NACI) Immunization Guide Chapter on Influenza and Statement on Seasonal Influenza Vaccine for 2015–2016 states “regarding administration of influenza vaccine to egg allergic persons, after careful review, NACI has concluded that egg allergic individuals may be vaccinated against influenza using trivalent influenza vaccine (TIV) without prior influenza vaccine skin test and with the full dose, irrespective of a past severe reaction to egg and without any particular consideration, including immunization setting [69].

### Measles Mumps and Rubella (MMR) vaccines

Most cases of anaphylaxis associated with MMR vaccines have been traced to the content of gelatin, which is used as a stabilizer. Reports of anaphylaxis following MMR have been reported for several decades, but the highest rate occurred prior to 1998, when the vaccines contained 0.2 % gelatin, with most reports coming from Japan. Nakayama et al. reported 366 cases of clinical reactions to MMR, of which 34 were anaphylaxis, 76 urticaria, and 215 cases had non-urticarial generalized eruption, while 41 had local reactions only. When serum was available, IgE antibodies to gelatin were detected in 25/27 (93 %) of those with anaphylaxis, 27/48 (56 %) of those with urticaria, 8/90 (9 %) of those with a generalized eruption, 0/41 with a local reaction only, and 0/29 control subjects [55] (C). Dramatic decreases in anaphylaxis/allergic reactions to live measles vaccines were observed in Japan immediately after each manufacturer marketed vaccines that were gelatin-free or contained a hypoallergenic form of gelatin. Since the end of 1998 reports of anaphylaxis/allergic reactions to live measles vaccines had almost disappeared [70, 71]. (D) D’Souza et al. reported adverse events following immunization to MMR in a review of the Measles Control Campaign (MCC) conducted in Australia from August to November 1998. There was only one anaphylactic reaction, giving a rate of 0.06 per 100,000 doses administered. The combined rate for anaphylaxis and allergic reactions was 1.06 per 100,000. The authors concluded that the benefits of the MCC far outweighed the risks of serious adverse events associated with immunization [72] (D).

In a separate report from VAERS, the rate of anaphylactic reactions reported after measles virus-containing immunization in the United States between 1991 and 1997 was 1.8 per one million doses distributed. Cases of anaphylaxis reported to VAERS during this time period were identified retrospectively and 57 subjects were recruited into a follow up study to investigate allergenic sensitization in relation to the event. Self-reported history of food allergy was present more frequently in the interviewed study subjects compared with controls who had also received vaccine without clinical reaction. Serum IgE analysis on 22 subjects showed that six (27 %) tested positive for anti-gelatin IgE, and none of 27 controls tested positive for anti-gelatin IgE. The levels of IgE antibody against egg and against all three viral antigens did not differ among study subjects and among controls [57] (D).

Concerns regarding risk of allergic reaction following MMR immunization of subjects who have clinical allergy to egg have been laid to rest. The manufacture of vaccines containing live virus produced in chick embryo cultures (measles and mumps) and human diploid cell culture (rubella) has resulted in a vaccine that contains no, or at most picogram quantities of egg protein, insufficient to cause an allergic reaction [73, 74]. In addition to those reports mentioned above, this has been confirmed in Iran [75] (D), Denmark [76] (D), Spain [77] (D), Finland [78] (D), and the United States [79, 80] (C or D). Persons with egg allergy can safely receive measles vaccine or MMR.

Minor allergic reactions with MMR vaccine are also infrequent. A prospective review of patients referred to an emergency department vaccination service in Dublin, Ireland included all referred cases for immunization from January 1, 2006 through December 31, 2010. Of the total 446 vaccines administered during the study period, 310 (69.5 %) were MMR. The majority of cases (261/310, 84.2 %) had been referred from the community for suspected egg allergy. Only six patients (1.3 %) experienced an immediate reaction to the vaccine and all reactions were minor [81] (D).

### Varicella vaccine

Varicella vaccines contain an attenuated live strain of varicella virus (Oka) combined with other components, including gelatin as a stabilizer. From May 1, 1995 through April 30, 1999, when over 16.1 million doses of Varivax (Merck) were distributed, a post-marketing safety study reported a total of seven cases of anaphylaxis in children ages 3 to 8 years. All but one occurred shortly after vaccine administration. Symptoms consisted of wheezing, stridor, swollen lips, urticaria, hypotension, coughing and itching. All affected were treated appropriately and recovered. In addition, there were 1349 cases of post-immunization rashes of which 4 % were classified as consistent with hypersensitivity [82] (D).

A separate post-licensure study of the VAERS database from March 17, 1995 through July 25, 1998 revealed 6574 case reports of adverse events after varicella immunization, a rate of 67.5 reports per 100,000 doses distributed. Approximately 4 % of reports were categorized as serious, including 14 deaths. The most frequently reported were rashes, possible vaccine failures and injection site reactions. There were 30 cases of reported anaphylaxis, none of which resulted in fatality [83] (D).

Similar to reports from Japan implicating the gelatin ingredient of MMR vaccine as a potential trigger for anaphylaxis, Sakaguchi et al. reported that anaphylaxis following administration of the varicella vaccine was associated with IgE antibody directed toward the gelatin component [84] (D). The estimated incidence of severe anaphylaxis associated with varicella vaccine from 1994 to 1996 in Japan was 10.3 cases per million doses of vaccine administered [85] (D). Ozaki et al. reported a rate of 28 serious anaphylactic reactions and 139 non-serious allergic reactions following gelatin-containing varicella vaccine from 1994 to 1999, when 1.41 million doses of varicella vaccine were distributed in Japan. All nine sera available from children with anaphylaxis were found to test positive for anti-gelatin IgE, whereas 55 of the 70 available sera from children with non-serious allergic reactions were positive. Conversely, there were no cases of anaphylaxis and only five cases of non-serious allergic reactions from 1999 to 2000 when 1.3 million doses of gelatin-free varicella vaccine were distributed [86]. The authors concluded that the newer vaccine was safe and also provided data that the immunogenicity was comparable to the earlier gelatin-containing vaccine [86] (D).

### Japanese encephalitis vaccine (JE-VC)

Vaccination is the single most important measure in preventing this disease. In March 2009, the U.S. Food and Drug Administration (FDA) licensed an inactivated, Vero cell culture-derived JE-VC (Ixiaro®) for use in adults. The vaccine replaced the prior Japanese Encephalitis Vaccine (JEV) that was derived from mouse brain and was licensed based on clinical trial safety data in 3558 JE-VC recipients.

A summary of the adverse events reported to VAERS for adults (≥17 years) who received JE-VC from May 2009 through April 2012 was recently published and included data on 275,848 JE-VC doses distributed [87]. Over the 3 year period, 42 adverse events following vaccination with JE-VC were reported to VAERS for an overall reporting rate of 15.2 adverse events per 100,000 doses distributed. Of the 42 total reports, five (12 %) were classified as serious for a reporting rate of 1.8 per 100,000 doses distributed; there were no deaths. Hypersensitivity reactions (N = 12) were the most commonly reported type of adverse event, with a rate of 4.4 per 100,000 doses distributed; no cases of anaphylaxis were reported. Three adverse events of the central nervous system were reported (one case of encephalitis and two seizures) for a rate of 1.1 per 100,000; all occurred after receipt of JE-VC with other vaccines. In conclusion, these post-marketing surveillance data suggest a good safety profile for JE-VC consistent with findings from pre-licensure clinical trials [87].

The newer inactivated Vero cell culture derived JE-VC vaccine does not contain potential mouse brain antigens nor gelatin as did the older vaccine, but does contain some protamine sulfate from the virus preparation step that requires protamine sulfate treatment to remove contaminating DNA and proteins. Protamine has been characterized as an allergen in the context of insulin allergy with protamine specific IgE contributing to the reactions [88]. Clinical trials safety data (less than 5000 vaccinees) did not show the serious systemic hypersensitivity reactions described with the older vaccine. Adverse events consistent with systemic hypersensitivity were observed at similar frequencies in recipients of the new vaccine (3.5 %) and the placebo (3.7 %) group. The placebo contained phosphate buffered saline and alum adjuvant so it was not an “inert” placebo. While studies to date suggest reduced risk of hypersensitivity reactions with the gelatin free newer vaccine, the actual incidence of potentially IgE-mediated reactions remains undefined. The package insert includes a caution in the setting of prior JEV reaction history and a documented hypersensitivity to protamine. Evaluation of future vacinees with serious immediate hypersensitivity reactions merit consideration of protamine as a relevant allergen [89, 90].

### Rabies vaccine

From October 1997 through December 2005, the Vaccine Adverse Event Reporting System (VAERS) received 336 reports of AEFIs to the purified chick embryo Cell (PCEC, RabAvert) vaccine, 20 of which were classified as serious, following vaccination in the U.S. Of the 20 serious AEFIs, three were classified as possible anaphylaxis. Most reported AEFIs are non-serious and consistent with pre-licensure safety data [91].

Reactions to the human diploid rabies vaccines were also reported from Poland [3]. In 289 patients receiving rabies diploid vaccine produced by Merieux, postvaccination reactions (14 %) included mainly local reactions with reddening, edema and pain at the injection site. These changes were short-lasting and resolved spontaneously in most cases. Systemic reactions included mainly fever with malaise (2 %), headaches and low mood (1.7 %). These reactions were also short-lasting and left no sequelae. Allergic reactions of the type of hyperergic purpura and urticaria were found in only isolated cases (0.3 %) [92].

### Tick-borne Encephalitis (TBE) vaccine

TBE vaccines target members of the virus family Flaviviridae that is one of the major human pathogenic flaviviruses causing potentially serious neurologic disease via three subtypes (European, Far Eastern and Siberian). The disease burden related to this pathogenic virus group continues to be of great concern [93, 94]. The TBE vaccine is not licensed in the US but is widely used in western and central Europe with over 100 million doses administered between 1980 and 2010 and major success in preventing TBE viral infections [95]. The safety surveillance experience has been reassuring. Immediate hypersensitivity reactions and anaphylaxis have not been reported as a post-marketing safety surveillance concern. In a PubMed search in April of 2015, only two publications can be found describing gelatin-induced urticaria and anaphylaxis (all associated with the older formulation). For post marketing surveillance of immediate allergic reactions, only one publication in 2004 reported a frequency of two per 100,000 doses with presumed linkage to the polygeline constituent. The newer vaccine introduced in 2002 (without polygeline for pediatric populations) demonstrated “no serious or unexpected adverse events related to vaccination were reported … more than 3000 voluntary subjects” [96]. As discussed above with Japanese encephalitis vaccine, whether or not protamine may become a clinically important allergen for susceptible individuals remains to be seen [97]. Finally, the package insert for the Canadian licensed vaccine states that "In the large clinical trials conducted to date, there were no reports in adults or children of serious clinical events, such as seizures, or of systemic allergic reactions, considered to be causally related to the vaccination." [98].

## Allergic reactions to vaccine components

Vaccines contain whole organisms or parts of organisms and/or inactivated toxins (toxoids) that induce protective immune responses. These vaccine antigens rarely, if ever, are the cause of hypersensitivity reactions. Recently, the mutant, non-toxic form of diphtheria toxin (CRM (197)), used as a carrier protein in Prevnar-13, was implicated as a cause of anaphylaxis in a 12 month old infant [99] (D). CRM (197) had previously been implicated as the allergen in a reaction to a Hib conjugate vaccine [100]. Other vaccine components that can induce allergic responses include residual media used to grow the organisms (e.g. yeast), adjuvants (e.g. aluminum salts), stabilizers (e.g. gelatin), antibiotics, preservatives (e.g. thimerosal) and trace amounts of latex from vaccine vial stoppers or syringe plungers in some vaccines (Table 2) [101, 102]. A complete list of all vaccine components that could be potential allergens can be found at the website of the Institute for Vaccine Safety of the Johns Hopkins University Bloomberg School of Public Health [103]. Many of these components are present in small amounts that are usually insufficient to induce allergic reactions in most individuals with possible hypersensitivity to the component. However, individuals with unusually high levels of IgE antibody can theoretically react to very small amounts of these antigens and develop severe reactions, including anaphylaxis.

**Table 2:** Recommended approach to patients with possible allergies to components of vaccines

Component	Vaccines	Recommendation
Egg	MMR	Give vaccine in usual manner without special precautions
	Influenza	Give vaccine in usual manner without special precautions
	Yellow Fever	Skin test with vaccine and if positive, administer in graded doses under observation
Gelatin	See Table 4	Skin test with vaccine and if positive, administer in graded doses under observation
Milk	DTaP	Give vaccine without special precautions
	Tdap	
Yeast	Hepatitis B	Skin test with vaccine and if positive, administer in graded doses under observation
	Quadrivalent HPV	
Latex	http://www.cdc.gov/vaccines/pubs/pinkbook/downloads/appendices/B/latex-table.pdf. Also, see [116].	Give vaccine without specific precautions

### Residual media

Residual small amounts of media to grow organisms are often found in both inactivated and live vaccines. For example, viruses are grown in cell lines. No intact cells from these cell lines persist in live or inactivated vaccines, and purification removes most of the cellular material, but it is impossible to remove all of the components.

### Adjuvants

Adjuvants are used to enhance the immune response to vaccines. Aluminum hydroxide and aluminum phosphate are the most common adjuvants used in vaccines. No immediate hypersensitivity reactions have been documented due to these adjuvants. However, contact allergy and small granulomas or nodules with persistent urticaria at the site may occur following aluminum containing vaccines and were observed in 38 of 4758 (0.83 %) prospectively followed children [104]. These urticarial granulomas usually persist for several months and rarely up to several years. Follow up 5 to 9 years after initial diagnosis in affected children revealed that the majority of children were no longer positive to aluminum contact allergy testing [105]. Larger recurrent nodules at the sites of injection of aluminum containing vaccines have been reported rarely and have resulted in biopsies to rule out tumors in predisposed individuals [106]. An increased rate of anaphylaxis and other immediate hypersensitivity reactions was reported in Canada associated with an AS03 (trade name for a squalene-based immunologic adjuvant used in various vaccine products by GlaxoSmithKline) adjuvanted pandemic H1N1 influenza vaccine [107]. A case–control study revealed higher rates of food allergy in affected individuals, but no evidence that the reactions were due to this adjuvant has been provided [108]. No increased risk of allergic reactions was noted in a systematic review of the safety of the MF59 (trade name for a squalene-based immunologic adjuvant by Novartis) adjuvanted influenza vaccine in children used in Europe [109]. This vaccine has been licensed for use in persons ≥ 65 years of age in the U.S. and there is no indication of an increase in reports of allergic reactions in clinical trials in the elderly to date [110].

### Antimicrobial agents

Gentamycin, tetracycline, neomycin, streptomycin, and polymyxin B are used during the production process for vaccines to prevent growth of bacteria or fungi [103]. Although most of these antimicrobials are removed during the purification process, trace amounts may be present in some vaccines. These antimicrobial agents can cause contact or rarely systemic hypersensitivity reactions when used in clinical settings at therapeutic doses (e.g. treatment of an infection). However, allergic reactions associated with the trace amounts present in vaccines have not been well documented [111].

### Preservatives

Thimerosal and 2-phenoxyethanol are used in multidose vials of vaccines to prevent bacterial growth. Thimerosal was used in several vaccines used in the United States until 2001, but was removed as a preservative in vaccines used in young infants as a precautionary measure because of theoretical concerns about mercury toxicity [102]. Some multi-dose vials of inactivated influenza vaccines contain thimerosal and trace amounts may be found in some other vaccines where thimerosal was used during the production process, but most was removed from the final product. Thimerosal in vaccines has been associated with contact allergy and rarely with systemic allergic reactions [112, 113]. 2-Phenoxyethanol and phenol have not been associated with immediate hypersensitivity reactions.

### Latex

Natural latex can cause immediate hypersensitivity reactions, including anaphylaxis [114]. Latex is present in the rubber stoppers on some vaccine vials, and on the plungers in some prefilled vaccines syringes (see Table 2). There are reports of immediate hypersensitivity reactions to latex in this setting, but in most instances, specific studies have not been done to determine that latex was the cause of the immediate hypersensitivity reaction [43, 115]. Nevertheless, patients with severe latex allergy should avoid vaccines packaged with latex-containing stoppers and syringe plungers if possible. Alternative vaccines without the risk of exposure to natural latex may be available. Synthetic latex which is not allergenic, has replaced natural latex in most products. A list of vaccines that contain natural latex in the packaging can be found in the index of the CDC Pink Book [116].

## Approach to the patient with a history of an allergic reaction to a vaccine

**Fig. 1:**
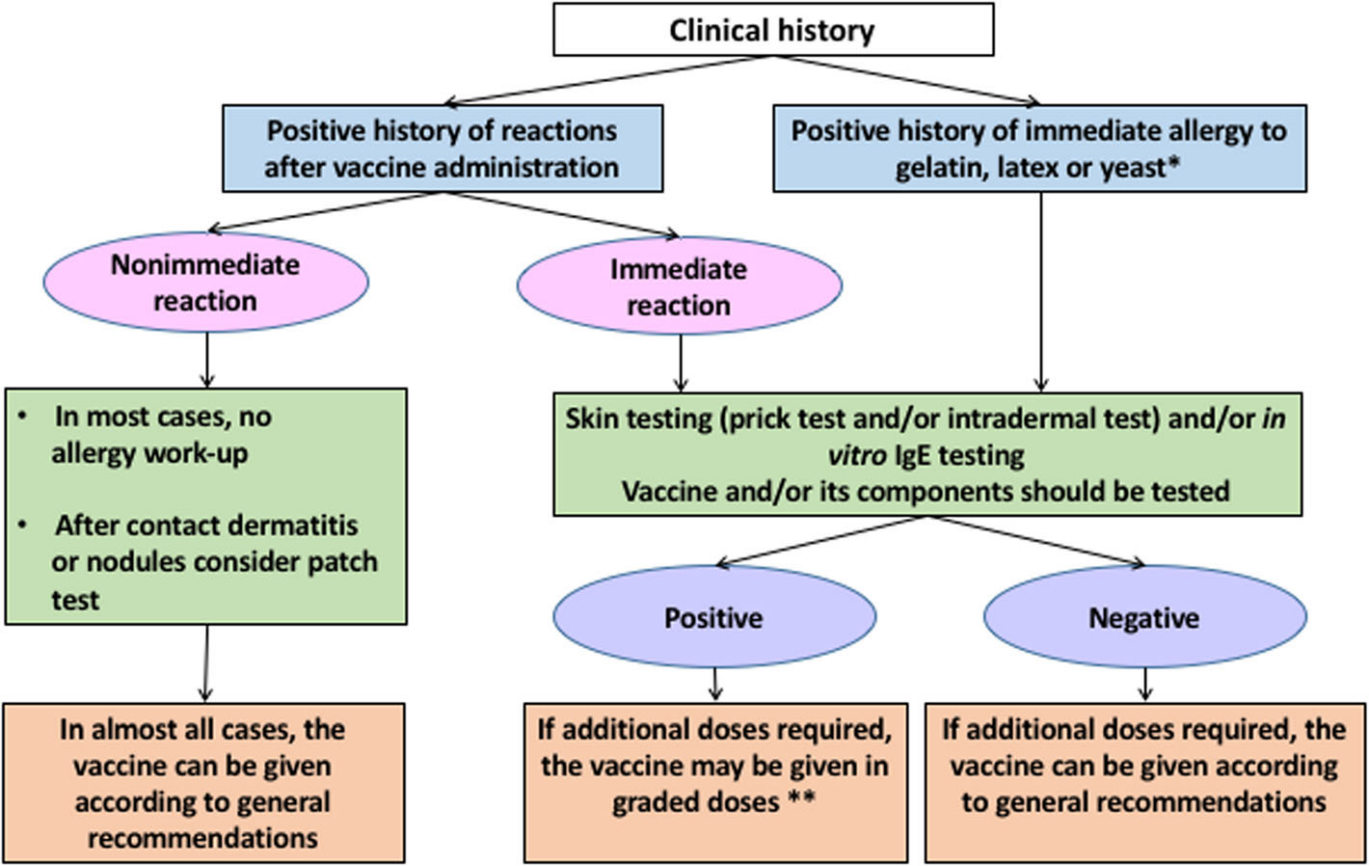
Management of patients with suspected hypersensitivity to a vaccine and patients with known allergy to a vaccine component (modified from Caubet et al. 2014; Printed with permission of Wiley) [120]. *For egg allergic patients, see text (Approach to the patient with possible allergies to foods or other materials that may also be components of vaccines or vaccine packaging). **For patients with a positive skin test to a vaccine, consider risk benefit analysis based on serologic evidence of current immunity and level of risk for target disease. See Wood et al. [119]

Several excellent practice parameters, reviews, and guidelines have been published describing the clinical management of patients with suspected vaccine allergy [4, 117–120]. The approach suggested by Caubet and colleagues [120] is reproduced, with minor modifications,  here in Fig. 1. Caveats that may alter management for specific patients are mentioned in the legend to Fig. 1 and are discussed in more detail by Wood et al. [119] and Kelso et al. [4].

### Approach to the patient with concerns regarding possible allergic reactions to vaccines

Some recommendations may change so the reader is encouraged to access the most up to date information whenever possible, such as from the Centers for Disease Control (www.cdc.gov/vaccines). Investigation of allergic reactions following the receipt of multiple vaccines simultaneously and/or combined vaccines is increasingly common and can be challenging. If serologic or skin testing are indicated the investigator may choose to prioritize the evaluations based on what they suspect to be the most likely allergens. When proceeding to the administration of additional doses of indicated vaccines, the investigator will need to assess each vaccine separately when possible. Conjugate polysaccharide-protein vaccines may require investigation of the proteins that are conjugated to the polysaccharides as well as other vaccine components as the plain polysaccharides are less likely causes of allergic reactions.

Most questions about vaccine allergy result from two general concerns. The first relates to patients who have had a possible reaction to prior vaccination, while the second relates to patients with a known allergy – such as egg allergy – that might put them at risk for specific immunizations (see below under the heading "Approach to the patient with possible allergies to foods or other materials that may also be components of vaccines or vaccine packaging"). Here we will focus on the patient presenting with concerns regarding a suspected reaction to a prior vaccine. The specific approach to these patients needs to carefully consider several key questions:

1. Was the reported event consistent with an IgE mediated allergy in terms of signs, symptoms, and timing? For example, the patient with a history of urticaria, angioedema, and respiratory distress occurring five minutes after vaccine administration is very different from the patient experiencing a non-specific rash 24 h after the vaccine was given (See Definitions, above).
2. Has the patient experienced a documented or suspected anaphylaxis or rash to any prior vaccines? If so, this might help to focus the evaluation on specific vaccine constituents that are common among the vaccines suspected of causing reactions.
3. Will the patient need additional doses of this vaccine or other vaccines with common constituents? Even if the patient will not need additional doses of the vaccine, an allergic reaction could indicate hypersensitivity to a vaccine component that might be in other vaccines the patient will receive. Thus, a thorough evaluation is needed even if no further doses of the suspect vaccine are required.

With these questions in mind, each patient can then be approached individually using a combination of clinical assessment, laboratory testing, and cautious re-administration of necessary immunizations.

### Clinical assessment

The clinician should first decide if future doses of the vaccine are truly needed. This assessment needs to consider the risk of re-vaccination against the risk of acquiring the vaccine preventable disease and of acquired disease severity. Some vaccines may be considered less important than others based upon the likely risk of exposure and presence of underlying risk factors. Since many vaccines are given as a series, some individuals may mount protective responses from the doses already administered and fewer than the recommended number of doses may produce lasting immunity. It may therefore be a reasonable option to measure and monitor IgG titers to assess the level of protection and the need for future doses, recognizing that antibody levels are not a useful measure of protection for all vaccines and that immunity might wane over time.

### Allergy testing with vaccines and vaccine constituents

If it is determined that additional doses of a vaccine should be administered, skin testing with the vaccine and/or vaccine constituents should be performed. This process may be relatively simple if only a single vaccine antigen was administered or far more complicated if multiple vaccines or multivalent vaccines (e.g. MMR) were given at the same visit, which is certainly the norm for the typical pediatric encounter.

A number of approaches to vaccine skin testing have been suggested but current guidelines recommend that testing be initiated with a prick skin test to the full strength vaccine, unless the patient has a history of severe anaphylaxis in which case it is appropriate to dilute the vaccine 1:10 or even 1:100 to initiate prick skin testing [4, 118] (D). If the prick skin test with full-strength vaccine is negative, an intradermal test with the vaccine diluted 1:100 should then be performed. All tests need to be interpreted carefully with appropriate positive and negative controls, recognizing that falsely positive skin test results may occur. These may be the result of true but clinically irrelevant IgE responses or to irritant effects of the vaccine. A case control study of a child with a history of anaphylaxis to the 23-valent pneumococcal vaccine positive skin tests and in vitro IgE tests to the whole vaccine, included nine controls [121] (C). In one study irritant reactions were common at concentrations of 1:10 or undiluted vaccines, especially with influenza, MMR, and varicella vaccines [122]. At the 1:100 concentration, rates of irritant reactions were far less common with the most frequent being 5 % for DT and DTaP and 15 % for influenza. It is also important to recognize that delayed responses (12–24 h) to vaccine skin tests are common, most likely representing previously established cell-mediated immunity, or immune complex formation in patients with high titers of antibody to vaccine components [123] (D), and should not raise concern in the evaluation of IgE-mediated vaccine allergy [122].

If the suspected vaccine contains specific constituents known to be potentially allergenic, testing should also be conducted for those components. These primarily include egg (for reactions to yellow fever or influenza vaccines), gelatin (see Table 3 for the gelatin content of specific vaccines), latex, and yeast. Skin test reagents for egg and yeast are commercially available. Prick skin test solutions for gelatin can be prepared by dissolving one teaspoon of gelatin powder in 5 mL of normal saline. Skin test extracts for latex are commercially available in many countries but not in the United States. In addition to skin testing, in vitro testing for allergen-specific IgE is available in most commercial laboratories for egg, gelatin, latex, and yeast. For gelatin, it is important that assays for both porcine and bovine products be conducted.

**Table 3:** Gelatin-containing vaccines approved for use in the United States (black type) and Europe (blue type) 2015

Vaccine	Gelatin content
Influenza (Fluzone [only in standard dose trivalent IM], Sanofi Pasteur)	250 micrograms per 0.5 ml dose
Influenza (FluMist, MedImmune Vaccines)	2000 micrograms per 0.2 ml dose
Influenza (Fluenz Tetra, MedImmune LLC)	Unspecified amount^a^ hydrolyzed gelatin, type A per 0.2 ml dose
Measles, Mumps, Rubella (MMRII, Merck)	14,500 micrograms per 0.5 ml dose
Measles, Mumps, Rubella, Varicella (M-M-RVAXPRO, Sanofi Pasteur)	Unspecified amount^a^ hydrolyzed gelatin per 0.5 ml dose
Measles, Mumps, Rubella, Varicella (ProQuad, Merck)	11,000 micrograms per 0.5 ml dose
Measles, Mumps, Rubella, Varicella (ProQuad, marketed in Europe by Sanofi Pasteur)	Unspecified amount^a^ hydrolyzed gelatin per 0.5 ml dose
Rabies (RabAvert, Novartis)	12,000 micrograms per 1.0 ml dose
Typhoid Vaccine Live Oral Ty21a (Vivotif, Crucell)	Capsule
Varicella (VARIVAX, Merck)	12,500 micrograms per 0.5 ml dose
Yellow Fever (YF-VAX, Sanofi Pasteur)	7,500 micrograms per 0.5 ml dose
Zoster (ZOSTAVAX, Merck)	15,580 micrograms per 0.65 ml dose
Zoster (ZOSTAVAX, marketed in Europe by Sanofi Pasteur)	Unspecified amount^a^ hydrolyzed gelatin per 0.65 ml dose

^a^Information provided in European Public Assessment Reports (EPAR) Product Characteristics documents do not specify quantities for excipients

Examples of skin and serologic testing that would be appropriate in the evaluation of suspected reactions to specific vaccines are presented in Table 4.

**Table 4:** Examples of testing used to assess specific vaccines suspected of causing allergic reactions

Vaccine	Skin testing	In vitro IgE testing
DTaP, Td, Tdap	DTaP, Td, Tdap,Tetanus toxoid, Gelatin, Milk	Gelatin, Milk
Hepatitis B	Hepatitis B, Yeast	Yeast
Influenza	Influenza, Egg, Gelatin	Egg, Gelatin
MMR	MMR, Measles, Mumps, Rubella, Gelatin	Gelatin
Varicella or Zoster	Varicella or Zoster, Gelatin	Gelatin
Yellow fever	Yellow fever, Egg, Gelatin	Egg, Gelatin
• Whenever possible, the same vaccine from the same manufacturer that was given at the time of the reaction should be used for testing		

### Administration of vaccines to patients with a history of a suspected prior allergic reaction

If both skin and in vitro testing are negative, especially if the intradermal skin test to the vaccine is negative, the chance that the patient has an IgE-mediated allergy to the vaccine or to any vaccine constituent is very small. The usual dose of the vaccine can therefore be administered with at least a 30 min observation period after vaccination in a facility where anaphylaxis can be recognized and managed with epinephrine and other supportive treatments.

If skin or in vitro testing to the vaccine or a vaccine component is positive, alternative approaches to vaccination should be considered. However, if the vaccine is considered necessary – that is, the benefit of the vaccine clearly outweighs the potential risk of vaccine administration – it is usually possible to safely administer the vaccine using a graded dose protocol [4]. These decisions should be carefully considered on a case-by-case basis, recognizing that even administration using a graded dose protocol still carries a threoretical risk of anaphylaxis. This should be conducted with informed consent and only in a setting prepared to treat anaphylaxis. As mentioned earlier, an overall approach to the patient with a suspected allergic vaccine reaction is presented in the algorithm in Fig. 1.

## Approach to the patient with possible allergies to foods or other materials that may also be components of vaccines or vaccine packaging

The most common situation that involve allergists being asked to evaluate patients is one in which a patient has a suspected allergy to an ingested substance (e.g. egg, milk, gelatin) or other allergen (e.g. latex) that is also a constituent of a vaccine. In some circumstances, pre-existing allergy to a vaccine component has been demonstrated to be the cause of anaphylactic reactions to vaccines containing the component (e.g. gelatin). However, allergy to components of vaccines has been suspected or demonstrated to be the cause of allergic reactions to vaccines only in very rare circumstances. Recommendations are outlined below.

### Eggs

Asking patients whether or not they are allergic to eggs is an adequate screen for egg allergy [124]. Most patients who would have a reaction to the ingestion of egg cooked by standard means, tolerate egg-containing baked goods without reaction. Such patients would still be considered egg-allergic for purposes of vaccine risk assessment [124]. Rare patients may be allergic only to heat-labile egg proteins (raw egg) and might not think of themselves as being egg-allergic [125].

The measles and mumps components of the measles-mumps-rubella (MMR) vaccine [126], and one type of rabies vaccine [127] are grown in chick embryo fibroblast cultures. A report of anaphylactic reactions to MMR vaccine in two egg-allergic children led to the notion that the vaccine contains egg protein that could cause reactions in egg-allergic recipients [128]. The authors stated that an assay of the vaccine had shown it to contain 1 ng of ovalbumin [128], although a previous study was unable to detect ovalbumin [129] and a subsequent study was able to detect only "37 pg of ovalbumin-like material" [79]. Even if the vaccine contains measurable amounts of ovalbumin or cross-reacting proteins, these reported amounts would be too small to elicit allergic reactions [130]. Numerous studies have demonstrated the safety of MMR in large numbers of egg-allergic children [80, 131] (C). Thus, egg allergy is no longer considered a contraindication to the administration of MMR vaccine and recipients need not be screened for egg allergy [7, 132]. As described below, most anaphylactic reactions to the MMR vaccine have been attributed to gelatin allergy.

Most injected inactivated influenza vaccines (IIV) and the intranasally-administered live attenuated influenza vaccine (LAIV) are grown in eggs and contain measurable amounts of ovalbumin [124]. Egg allergy was considered to be a contraindication to the administration of these vaccines for many years; however we now know that these patients can safely receive influenza vaccines (See previous section "Influenza vaccine") [133]. The incidence of anaphylaxis after influenza vaccine is estimated to be about one per million doses [61, 134, 135]. However, the egg allergy status of the patients who have had such reactions is unknown and anaphylactic reactions are also reported after administration of egg-free influenza vaccine [136]. Twenty-eight studies specifically addressing the safety of the administration of IIV to egg-allergic recipients have collectively evaluated over 4300 subjects, including over 650 with histories of anaphylactic reactions to the ingestion of egg, with reaction rates similar to non-egg allergic recipients and without any serious reactions [130]. This is likely because the vaccines marketed in the United States do not contain sufficient ovalbumin to trigger a reaction even in highly egg-allergic subjects. All influenza vaccines currently available in the United States and Europe contain less than 1 mcg of ovalbumin per dose [124, 137, 138]. However, for many of the vaccines available in other areas of the world, the egg protein content is unknown.

Given the extensive body of evidence demonstrating the safety of IIV in egg-allergic recipients, statements endorsed by the United States Joint Task Force on Practice Parameters and the Canadian National Advisory Committee on Immunization have been published [69, 139]. These documents state that any age approved influenza vaccine can be used in any patient irrespective of egg allergy status and that special precautions are not required [69, 139] (A). As above, anaphylaxis can rarely occur in any patient after the receipt of any vaccine and providers should be prepared to recognize and initiate treatment for such reactions [16].

At the time these recommendations were made, studies had not addressed the safety of LAIV in egg-allergic patients. However, three recent studies evaluated the administration of LAIV in (collectively) 955 egg-allergic children with no systemic allergic reactions [140–142] (Des Roches C; Turner (BMJ) B; Turner (JACI) B). Thus, LAIV can also be administered safely without special precautions to egg allergic recipients. In 2015 the ACIP did not recommend use of LAIV in persons with egg allergy because of a lack of data [143]. For adults with a history of anaphylaxis following egg ingestion, recombinant IIV was recommended and the guidelines called for observation for at least 30 min after IIV. These guidelines are under active review and should be revised soon based on the recently completed studies indicating LAIV can be used in egg allergic individuals and special precautions may not be needed following IIV.

Yellow fever vaccine is prepared in chicken embryos [144] and contains measurable amounts of ovalbumin [129]. The vaccine may also contain chicken proteins [145]. Anaphylactic reactions have been reported after receipt of yellow fever vaccine, but the egg allergy status of these patients is not known [146]. There are no studies evaluating the administration of yellow fever vaccine as a single dose in the usual manner to egg-allergic recipients and thus it is unknown whether or not this would induce allergic reactions. The package insert describes a protocol for vaccine skin testing in egg-allergic or chicken-allergic recipients [144]. A prick skin test is performed with the vaccine diluted 1:10, and if negative, an intradermal skin test is performed with the vaccine diluted 1:100. If these skin tests are negative, the vaccine can be administered in the usual manner. If the skin tests are positive, the vaccine can be administered in graded doses under observation giving the following amounts at 15 min intervals: 0.05 mL of a 1:10 dilution and then using full strength vaccine, 0.05 mL, 0.10 mL, 0.15 mL and finally 0.20 mL. This or similar protocols have allowed egg-allergic patients to safely receive yellow fever vaccine [145, 147, 148].

### Gelatin

While simply asking patients if they are allergic to eggs is typically an adequate screen as above, the same may not be true for gelatin allergy. Many patients who have had anaphylactic reactions to gelatin-containing vaccines tolerate the ingestion of gelatin [149]. Presumably this is because ingestion allows the digestion of gelatin into smaller, less allergenic peptide fragments [150]. Thus, candidates for gelatin-containing vaccines should still be asked whether or not they are allergic to gelatin, and those who report such allergy should be evaluated prior to receiving such vaccines. However, when evaluating a patient who has suffered an apparent allergic reaction after receiving a gelatin-containing vaccine, the fact that the patient can ingest gelatin uneventfully does not exclude gelatin allergy as the cause of the vaccine reaction.

Gelatins used in foods and vaccines may be of bovine or porcine origin [149], which are extensively, but not completely, cross-reactive [149, 151, 152]. Serum specific IgE antibodies for both bovine and porcine gelatin are commercially available. Although there are no approved commercial skin test extracts for gelatin, a crude extract for a prick skin testing can be made by dissolving 1 level teaspoon (5 g) of any flavor of sugared gelatin in 5 mL of normal saline [117]. Consideration should be given to performing both of the in vitro tests and the in vivo test in patients with a history of allergic reactions to the ingestion of gelatin or the receipt of a gelatin-containing vaccine [4, 6].

Gelatin is added to many (mostly live attenuated viral) vaccines as a stabilizer (Table 4). It is the vaccine constituent that has been most convincingly demonstrated to be responsible for allergic reactions. The original case report described a patient who suffered an anaphylactic reaction after receipt of MMR vaccine and who had previously had allergic reactions after the ingestion of gelatin [152]. The only vaccine constituent to which she made IgE antibody was gelatin and RAST inhibition studies demonstrated cross-reactivity between food source gelatin and the pharmaceutical gelatin used in the vaccine. Subsequent studies demonstrated gelatin to be the culprit allergen in anaphylactic reactions to MMR [149, 153, 154], varicella [84, 155], Japanese encephalitis [156] and TBE vaccines [157]. In some countries, vaccine manufacturers have removed gelatin from vaccines or changed to a more thoroughly hydrolyzed, and thus less allergenic, gelatin and this has been associated with marked decreases in allergic reactions to these vaccines [71, 86, 158, 159].

In a patient with a history of an allergic reaction to the ingestion of gelatin or to the receipt of a gelatin-containing vaccine who requires additional doses of the same or another gelatin-containing vaccine, in vitro and in vivo gelatin tests should be performed as above. In addition, a prick skin test should be performed with the vaccine full-strength and if negative, an intradermal skin test should be performed with the vaccine diluted 1:100 [4, 119, 122]. If the skin tests are negative, the vaccine can be given in the usual manner and the patient observed for 30 min afterwards [4]. If the skin tests are positive and additional doses of the vaccine are required, the vaccine can be given in incremental doses under observation, prepared to treat an allergic reaction [4]. For example, for a vaccine where the volume of the normal dose is 0.5 mL, the following can be given under observation at 15 min intervals: 0.05 mL of a 1:10 dilution and then using full strength vaccine, 0.05 mL, 0.10 mL, 0.15 mL and finally 0.20 mL.

### Milk

Milk allergy is quite common, particularly in children. A case series has been published of eight children with severe milk allergy who had allergic reactions to DTaP or Tdap vaccines [160]. The children all had very high serum levels of milk-specific IgE antibody. Skin tests were not performed with the vaccines. However, although the specific lots of vaccines that caused the reactions were not available for testing, other lots of the same vaccines were assayed and found to contain nanogram quantities of casein. The bacteria used in preparation of these vaccines are grown in culture media that contains amino acids derived from casein [160]. A case series has also been published of four milk-allergic children who had allergic reactions after receiving a particular brand of oral polio vaccine containing alpha-lactalbumin [161]. The overwhelming majority of milk-allergic children receive these vaccines uneventfully. If milk allergy is responsible for these reactions, it likely involves the very rare coincidence of an exquisitely allergic patient and a particular lot of vaccine contaminated with larger milk peptide fragments [162]. Thus, no special precautions are required when administering vaccines to milk-allergic patients [162, 163]. However, should a milk allergic patient suffer an allergic reaction to one of these vaccines, the possibility of milk protein contaminating the vaccine should be considered.

### Yeast

The yeast *Saccharomyces cerevisiae* is commonly known as baker's yeast or brewer’s yeast. An occupational disease known as baker's asthma is usually due to allergy to cereal grains, [164, 165] but can very rarely be caused by allergy to *S. cerevisiae* [166]. This yeast is also a very rare cause of food allergy [167]. In addition, some patients believe they suffer from "yeast hypersensitivity syndrome", an ill-defined and unproven condition [168], and might also consider themselves allergic to yeast. Hepatitis B vaccines may contain viral proteins grown in *S. cerevisiae* and contain 1–5 % residual yeast protein (up to 25 mg per dose) [169, 170]. Quadrivalent human papillomavirus vaccine also contains residual yeast protein (less than 7 micrograms per dose) [171]. A review of 180,895 adverse event reports to VAERS, revealed 107 reports that mentioned a history of allergy to yeast and occurrence of symptoms after any vaccination [172]. Eighty-two of those 107 reports involved hepatitis B vaccine and 11 of those 82 described possible anaphylactic reactions. The other reports described common vaccine reactions such as injection site reactions or fever. Four reports described possible anaphylactic reactions in recipients reporting yeast allergy after receiving non-yeast-containing vaccines. Thus, both yeast allergy and adverse vaccine reactions attributable to yeast allergy appear to be exceedingly rare. A patient who reports yeast allergy should be carefully questioned about the nature of exposure and nature and timing of symptoms and undergo prick skin testing or serum specific IgE antibody testing with *S. cerevisiae* to reveal the rare patient who may have symptoms due to IgE-mediated yeast allergy. In such patients, it would seem prudent, prior to hepatitis B vaccination, to perform vaccine skin testing and, if positive, vaccine administration in graded doses as described above for gelatin containing vaccines or use a vaccine not grown in yeast.

### Latex

Potential exposure to latex in vaccines is related to the packaging, either the vial or syringe [173]. The "rubber" in vaccine vial stoppers or syringe plunger tips may be dry natural rubber (DNR) latex or synthetic rubber. Those made with DNR pose a theoretical risk to latex-allergic patients. However, unlike flexible latex products such as gloves and balloons from which latex allergen can be easily eluted [174], it is difficult to elute latex allergen from these molded rubber products [175]. A review of 167,233 adverse event reports to VAERS revealed 147 reports that mentioned a history of allergy to latex and occurrence of symptoms after any vaccination [115]. Twenty-eight of these 147 reports described possible allergic reactions in vaccine recipients, only two of whom were hospitalized and all recovered completely. The reports involved a wide variety of vaccines that may or may not have contained latex in the packaging. Thus, it appears that allergic reactions to vaccines caused by latex in the packaging are exceedingly rare. Therefore, no special precautions, aside from using non-latex gloves for the injection, are required when administering vaccines to latex allergic patients beyond the brief observation period recommended after the administration of any vaccine to any patient. However, as for any patient, providers should be prepared to treat unusual allergic conditions and reactions.

## Unmet needs

Allergic reactions to vaccines are infrequent but potentially life-threatening events that are poorly understood. For example, it may be possible to identify risk factors such as clinical history (e.g. concomitant illnesses), gender, specific genetic polymorphisms, and concomitant exposures that may work independently or in concert to increase risk of a reaction or a poor outcome if such a reaction takes place. In addition, the current evaluation of patients who have had a severe allergic reaction to a vaccine is quite cumbersome and would be expedited if dependable in vitro testing were available. A list of suggested future studies is presented in Table 5.

**Table 5:** Research priorities for allergic reactions associated with immunizations

1.	A case control study to evaluate risk factors including prior exposures to vaccines, foods, environmental factors, gender, and clinical history.
2.	Understanding genetic factors that predispose to allergic reactions and particularly anaphylaxis, following commonly used vaccines.
3.	Evaluation of a simplified checklist with illustrations of the steps that should be taken for a patient with suspect anaphylaxis coupled with the development of a standard small kit to be placed in immunization clinics with simplified instructions as to how to administer epinephrine, IV fluids, steroids, and antihistamines.
4.	Development of an in vitro assay (e.g. the basophil activation test (BAT) to evaluate for sensitivity to suspect allergens and thus, avoid the need for skin testing and trial doses of vaccines in people who have previously had an anaphylactic reaction.
5.	Development of an alternative to gelatin as a stabilizer in measles and varicella vaccines.

## Executive summary

Allergic reactions to vaccines are rare events and need to be distinguished from a variety of less important and more frequent adverse events following immunization. Given the large numbers of vaccines given worldwide, an international consensus for the evaluation and management of allergic reactions to vaccines as presented here is important. This document provides comprehensive and internationally accepted guidelines and access to on-line documents to help practitioners around the world identify allergic reactions following immunization. It also provides a framework for the evaluation and further management of patients who present either following an allergic reaction to a vaccine or with a history of allergy to a component of vaccines.

## Global implications for press releases

Allergic reactions to vaccines are rare occurrences but can be life threatening. Given the large numbers of vaccines given throughout the world, it is critical to reach an international consensus regarding the approach to patients with possible allergic reactions to vaccines and to patients with other allergic diseases who may have concerns about receiving specific vaccines. This International Consensus Document on “Allergic Reactions to Vaccines” achieves this goal.

## Abbreviations

AAAAI: American Academy of Allergy, Asthma, and Immunology
ACAAI: American College of Allergy, Asthma, and Immunology
ACIP: Advisory Committee on Immunization Practices
AEFI: Adverse event following immunization
CDC: Centers for Disease Control and Prevention
CENTRAL: Cochrane Central Register of Controlled Trials
CINAHL: Cumulative Index to Nursing and Allied Health
CISA: Clinical Immunization Safety Assessment network
DARE: Database of Abstracts of Reviews of Effects
DTaP: Diphtheria Tetanus and acellular Pertusis vaccine
DTP: Diphtheria Tetanus and Pertusis vaccine
EAACI: European Academy of Allergy and Clinical Immunology
EMBASE: Excerpta Medica dataBASE
FAAN: Food Allergy and Anaphylaxis Network
FDA: Food and Drug Administration
H1N1: the H1N1/09 influenza virus strain responsible for the 2009 flu pandemic
HHE: Hypotonic Hyporesponsive Episode
IIV: Inactivated Influenza Vaccine
IOM: Institute of Medicine
ORS: OculoRespiratory Syndrome
ICON: International CONsensus
JCAAI: Joint Council of Allergy, Asthma, and Immunology
JE-VC: Japanese Encephalitis Vaccine
LAIV: Live attenuated influenza vaccine
LAMV: Live attenuated monovalent vaccine
MCC: Measles Control Campaign
MMR: Measles Mumps Rubella vaccine
NACI: Canadian National Advisory Committee on Immunization
NIAID: National Institute of Allergy and Infectious Diseases
NIH: National Institutes of Health
TBE: Tick-borne encephalitis
TOXLINE: Toxicology Literature Online
TIV: Trivalent influenza vaccine
VAERS: Vaccine Adverse Events System
VICP: U.S. Vaccine Injury Compensation Program
VSD: Vaccine Safety Datalink
WAO: World Allergy Organization
WHO: World Health Organization

## Footnotes

1 All relevant references are stratified as having a level of evidence A-D (see Box 3 in [176]).
